# Pilot Evaluation of Sealer-Based Root Canal Obturation Using Epoxy-Resin-Based and Calcium-Silicate-Based Sealers: A Randomized Clinical Trial

**DOI:** 10.3390/ma15155146

**Published:** 2022-07-25

**Authors:** Minju Song, Min-Gyu Park, Sang-Won Kwak, Ruben H. Kim, Jung-Hong Ha, Hyeon-Cheol Kim

**Affiliations:** 1 Department of Conservative Dentistry, College of Dentistry, Dankook University, Cheonan 31116, Korea; minju81s@dankook.ac.kr; 2 Department of Conservative Dentistry, Dental Research Institute, School of Dentistry, Pusan National University, Yangsan 50612, Korea; hihoney22@naver.com (M.-G.P.); endokwak@pusan.ac.kr (S.-W.K.); 3 UCLA Jonsson Comprehensive Cancer Center, David Geffen School of Medicine at UCLA, Los Angeles, CA 90095, USA; rkim@dentistry.ucla.edu; 4 Department of Conservative Dentistry, College of Dentistry, Kyungpook National University, Daegu 41566, Korea; endoking@knu.ac.kr

**Keywords:** calcium silicate, root canal filling, sealer, sealer-based obturation

## Abstract

The purpose of this randomized controlled clinical trial was to assess the short-term clinical effectiveness of sealer-based root canal obturation using calcium-silicate-based sealer (CSBS) and epoxy-resin-based sealer (ERBS). A total of eighty patients (eighty teeth) were enrolled and seventy-two patients (seventy-two teeth) were randomly assigned into four different sealer groups: AH Plus (AH, Dentsply Sirona), ADseal (AD, Meta Biomed), CeraSeal (CS, Meta Biomed), or EndoSeal TCS (ES, Maruchi). At the first visit, root canal treatment was performed using ProTaper. Next, the NiTi file system and passive ultrasonic irritation was applied with 2.5% NaOCl. At the second visit, the teeth were obturated with a sealer-based obturation technique using the assigned sealer. The quality of filling obturation was evaluated with postoperative radiographs in terms of the presence of voids and sealer extrusion. The patients were recalled at 1-week, 1-month, and 3-month intervals, and pain levels were measured using visual analog scales (VAS), and clinical examination performed with percussion and palpation. Radiographs were also taken. The results were statistically compared by using the χ^2^ test or Fisher exact test. Among the 80 teeth included, 72 teeth received allocated intervention and 71 teeth were included in the analysis (98.6% recall). There was no significant difference among the sealers in void and sealer extrusion (*p* > 0.05). Postoperative pain was not indicated prominently regardless of the type of sealer. For a 3-month follow-up period, all cases were maintained successfully except for one case from AD. According to the findings, there was no significant difference between CSBS and ERBS in terms of postoperative pain or the healing process, and the variation in filling quality appears to be linked to the properties of each product.

## 1. Introduction

Since the mid-19th century, gutta-percha (GP) has been used as a root canal filling material after removing the diseased pulp tissues. GP itself cannot seal the canal completely; as such, it is always used with sealers to seal off the minor gaps between the GP and canal wall and irregularities of the root canal systems [1,2]. Sealer itself also has shortcomings, such as microleakage, dimensional stability, and biocompatibility; therefore, it is recommended to use as thin sealer as possible [3,4]. The warm vertical compaction or continuous wave of condensation technique using GP and epoxy-resin-based sealer (ERBS), the gold standard for root canal sealers, is the most popular and suitable technique for increasing the amount of GP and decreasing the amount of sealer in the root canal area.

Along the advancement in rotary nickel–titanium (NiTi) instruments, the single-cone technique using a matching single-cone has increasingly gained popularity owing to its simplicity, lesser technique-sensitivity, and short working time [5]. However, several studies reported that the single-cone technique showed inferior filling quality to other warm vertical obturation or pressured condensation techniques [6,7]. Such shortcomings are presumably due to the disparity between the round tapered GP and the irregular canal shape, resulting in a relatively larger amount of sealer used in the single-cone technique compared to other obturation techniques.

ERBS has been regarded as the gold standard for root canal sealers due to its resorption resistance and dimensional stability, but it does have drawbacks, including the possibility of mutagenicity, cytotoxicity, an inflammatory response, and hydrophobicity [8,9,10,11]. Recently, calcium-silicate-based sealer (CSBS) was introduced as an alternative to ERBS. CSBS has superior physicochemical and biologic properties. A growing body of evidence supports a notion that CSBS materials are biocompatible, stimulate biomineralization, have antibacterial activity, have a higher binding strength to dentin than traditional sealers, and provide a superior seal on par with ERBSs [3,4,12,13,14], suggesting that CSBS may potentially replace and minimize complications related to ERBS.

The first commercialized CSBS, iRoot SP (Innovative Bioceramix, Vancouver, BC, Canada), was developed in 2007 and demonstrated biocompatibility and hydrophilicity [13]. Since then, various sealers have been brought to the market, such as EndoSequence BC Sealer (Brasseler USA, Savannah, GA, USA), MTA Fillapex (Angelus Soluções Odontológicas, Londrina, Brazil), and so on. CeraSeal (Meta Biomed, Cheongju, Korea) is a premixed calcium-silicate-based material sealer composed of calcium silicates and zirconium oxide [14]. EndoSeal MTA (Maruchi, Wonju, Korea) has been marketed as a pozzolan-based MTA sealer [12]. With the specific chemical composition and manipulation method using a syringe, it has shown to have a superior filling ability, showing a low void percentage and lower solubility compared to conventional CSBS [15,16]. Both were marketed as injectable pastes that were premixed and ready to use. Although the physicochemical properties were evaluated and compared in many articles recently published [4,12,13,15], there is relatively little information in the literature on its clinical performance. Therefore, the purpose of this pilot study was to assess the short-term clinical effectiveness of sealer-based root canal obturation using ERBSs and CSBSs.

## 2. Materials and Methods

### 2.1. Study Design and Inclusion/Exclusion Criteria

This prospective clinical study was conducted for the patients who were recruited at a university dental hospital from November 2020 to October 2021. The study protocol of this project was approved by the Institutional Review for Research on Human Subjects (PNUDH-2020-045-MD), and informed consent was acquired from all participants.

The teeth were examined with periapical radiographs, periodontal probing, percussion testing, and sensibility assessment. The teeth diagnosed as asymptomatic irreversible pulpitis and/or pulp necrosis with/without chronic apical periodontitis were included in the study. Teeth with excessive mobility, a crack, radiographic evidence of internal/external resorption, or calcification of the pulp chamber or canals were excluded. Teeth diagnosed as acute periapical periodontitis or alveolar abscess were excluded. Patients who have systemic diseases (e.g., severe diabetes mellitus) that may have an effect on the outcome of root canal treatments and who need intentional endodontic treatment for a certain purpose were also excluded. C-shaped root canal, identified during shaping procedure, was excluded from this study as well.

### 2.2. Sample Size Calculation and Randomization

The sample size was determined using the G*power program that is commonly used to calculate the minimum required sample size for various statistical analyses (*t*-test, F-test, chi-square test, z-test, etc.) [17]. We obtained a total of 69 sample size with a significance level (α) of 0.05, power (1 − β) of 0.8, medium effect size (ω) of 0.4, and the degree of freedom (Df) of 3. The initial goal is to enroll a total of 83 subjects based on the assumption that 20% of the patients may fail to attend the follow-up. We determined that this number is appropriate by referring to previous similar studies [18,19].

**Figure 1:**
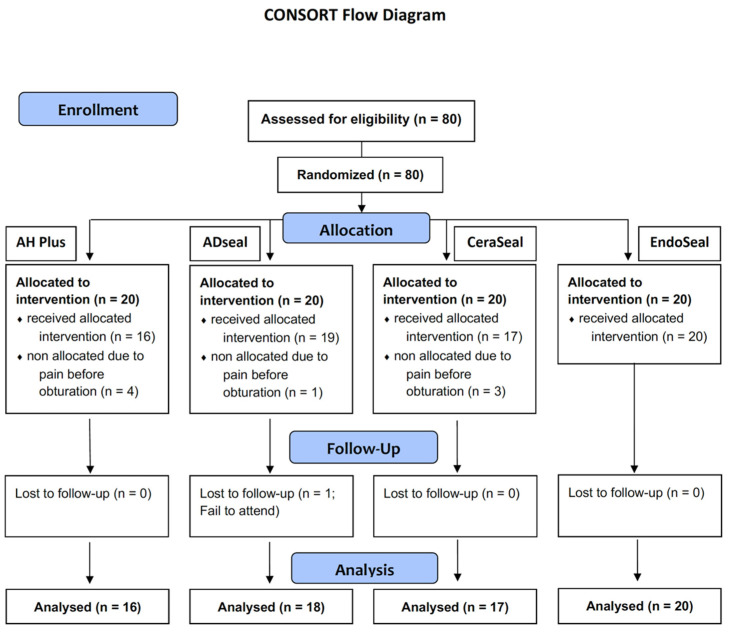
CONSORT flow diagram of this study.

Total 80 patients (80 teeth) were eligible for this study, and they were randomly assigned to the 4 root canal sealers used in this study (n = 20): AH Plus (group AH; Dentsply Sirona) and ADseal (group AD; Meta Biomed, Cheongju, Korea) as ERBS, and CeraSeal (group CS; Meta Biomed) and EndoSeal TCS (group ES; Maruchi, Wonju, Korea) as CSBS. The sealers were randomly applied to the teeth using pre-set order made by randomization (Figure 1). Because the handling properties of the sealers are different, the study was not able to perform in a blinded manner by the endodontist.

### 2.3. Treatment Procedure

A two-visit common root canal treatment was performed by the endodontist, who was experienced more than 20 years. On the first-visit, tooth was isolated with rubber dam after anesthesia. Access cavity was made after removal of caries dentin and old restoration if existed. The coronal canal was flared using OneFlare (MicroMega, Besançon, France), and glide-path was prepared using PathFile #1 (Dentsply Sirona, Ballaigues, Switzerland). Root canal was instrumented using ProTaper Next rotary NiTi file systems (Dentsply Sirona) with endodontic motor (E-connect S; Eighteeth, Changzhou, China), and final instrumentation sizes, X2, X3, X4, and X5, were decided according to the size of natural canals. During the canal preparation, copious amounts of sodium hypochlorite (NaOCl, 2.5%) were used for canal irrigation, and passive ultrasonic irrigation (PUI) was applied using UC-One (EP dent, Gimpo, Korea). After drying the canals with paper points (Meta Biomed), calcium hydroxide paste (Calcipex II; Nishika, Shimonoseki, Japan) was applied and access was temporarily restored using Caviton (GC, Tokyo, Japan).

On the second visit for root canal obturation, the patient was asked if any symptoms remained. Patients without any clinical symptoms were confirmed and continually included in this study. Teeth were isolated and temporary restoration was removed completely. Intracanal medication was also removed, and canal was irrigated thoroughly with NaOCl and ethylenediaminetetraacetic acid (EDTA) using PUI unit. After drying the canals with paper points, sealers were randomly assigned and delivered by using the premixed syringe and master GP cone for the groups CSBS and ESBS. For groups AD and AH, sealers were applied using master GP cone. The master cone was introduced to the working length. Then, multiple times pumping motion of the master GP cone was performed to improve sealer flow and remove entrapped air void. At the level of the canal orifice, the coronal part of GP was severed and compacted with hand plugger.

### 2.4. Clinical and Radiographic Evaluation

Postoperative radiographs were taken using the paralleling technique to evaluate the canal filling quality and sealer extrusion. From all root canals, the number of voids entrapping below the canal orifice was counted using the two different radiographs with a shifted direction. Sealer extrusion was counted per both canal and tooth.

Patients were recalled at around 1 week (7 to 10 days) after canal obturation for coronal restoration or core build-up for full coverage restoration. Then, patients were recalled at 1 month and 3 months to check for any unexpected postoperative symptoms and clinical signs (responses on percussion and palpation) or flare-ups related to the treated teeth. At every visit, subjective clinical symptoms were evaluated for the pain levels using visual analog scales (VAS), and for clinical examination with percussion and palpation. Radiographs were also taken at every visit to evaluate the resolution of apical radiolucency. In cases in which the patient has unexpected symptoms and pain during the follow-up periods, proper procedures were planned for retreatment.

### 2.5. Statistical Analysis

To analyze the frequencies of void and sealer extrusion according to the materials, a Pearson chi-square test or Fisher’s exact test was used with a significance level of 0.05. Statistical analyses were performed by using SPSS version 15.0 (SPSS Inc., Chicago, IL, USA).

## 3. Results

**Figure 2:**
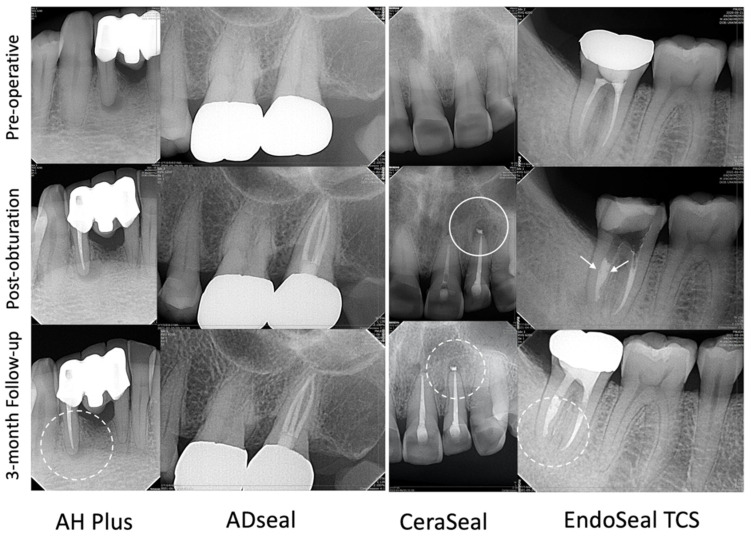
Representative cases obturated with 4 sealers used in this study. Radiographs of pre-operative, post-obturation, and 3-month follow-up from above to bottom. Dot circles show healing or healed apical lesion on 3-month recall. A case with CeraSeal shows sealer extrusion (full circle). A case with EndoSeal shows minimal void (white arrows, scored as 1) in the obturation.

Among the 80 patients (80 teeth) who were initially eligible for this randomized controlled trial, 72 patients (72 teeth) were included in this study, and one patient (one tooth) was dropped due to unavailability to follow up within 3 months (98.6% recall). Table 1 listed the distribution of the analyzed cases according to the sealer used. Representative case samples are presented in Figure 2.

From the radiographs taken after canal filling, ES (60%) and AH (56.3%) showed higher void numbers than AD (31.6%) and CS (29.4%) (Table 2). Sealer extrusion was found more frequently in AH (56.3%, 33.3%) and CS (58.8%, 29.7%) than ES (35%, 17.8%) and AD (21.1%, 12.2%) on evaluation per tooth and canal. However, there were no significant differences among the sealers in void and sealer extrusion (*p* > 0.05).

Postoperative pain was not remarkably different among the tested sealers (Table 3). All the groups indicated a similar postoperative pain level, ranging from 0 to 0.06 VAS at 1 week after canal filling, and did not show any change except for the AH group. The VAS in the AH was 0.06 at 1 week and increased to 0.13 at 1 month and 3 months. AD showed a positive response to percussion in only one case. Besides one case of AD, there was no case having a positive response to percussion and palpation at any assessed time interval.

For the 3-month follow-up period, all cases were maintained successfully except for one case from AD. AD showed one case of recurring sinus tract at one month after canal filling. Radiographic changes occurred in some cases having a periapical lesion in each group. AD showed the most regression of periapical radiolucency in three out of seven cases, followed by ES (four out of ten), AH (two out of six), and CS (two out of eight cases) (Table 4).

## 4. Discussion

The introduction of CSBS has been a key advance in the field of filling materials over the previous decade, as well as having brought a shift in the concept of obturation procedures [20]. Sealer-based obturation using CSBS is similar to the traditional single-cone technique in the aspect of using a pre-fitted single GP cone. However, there is a typical distinction in that the sealer is responsible for providing the seal as the main contents of the canal space [21]. To achieve a successful outcome of root canal therapy, CSBS should have an efficient clinical assessment, as well as excellent physicochemical and biological properties in vitro and in vivo. Therefore, this study assessed CSBSs in terms of filling quality, postoperative pain, and radiographic change compared with two different ERBSs. Considering that the canal obturation technique might be one of the affecting factors on the postoperative pain and sealer extrusion [21,22,23], sealer-based obturation was used for all types of sealers in this study.

To evaluate the quality of the canal filling, void formation and sealer extrusion (per canal) were investigated. The voids may act as a reservoir for bacteria, resulting in microleakage and endangering the long-term success of root canal therapy. Several factors, including the obturation technique, physical qualities of the materials (e.g., film thickness, flowability, and wettability), and the anatomical structure of the root canal system, might impact the void formation [24,25]. CSBS has been known to show comparable or superior flowability compared to the ERBS [26] and demonstrate less void during root canal filling [27]. However, this study showed a high percentage void in AH Plus and EndoSeal without showing the tendency according to the type of sealer. Previous research has shown that the incidence of voids is higher in oval root canals, especially when using the single-cone technique [28,29]. This study showed that void was found to be more common in molars (50%, 23/46 teeth) than premolar (38.5%, 5/13 teeth) or anterior (30.7%, 4/13 teeth) teeth. The void incidence seems to be more related to root canal anatomy rather than the type of sealer in this study as well [30].

In this study, we did not find any differences in sealer extrusion among the tested sealers, albeit with higher frequencies noted in AH Plus and CeraSeal (Table 2). Filho et al. reported that CSBS showed more sealer extrusion compared to ERBS [31], whereas Tan et al. showed that AH Plus is more related to cases with sealer extrusion rather than TotalFill BC, one of the CSBSs [23]. However, the clinical outcome is not associated with sealer extrusion [32,33]. Nonetheless, depending on the type of sealer used, it may trigger an adverse tissue reaction, such as an inflammatory or foreign body reaction [34]. Despite the fact that this is a rare occurrence, some research has suggested that root canal sealers may cause an inflammatory response and sensory nerve activation [35,36]. Given that the chemical composition of the extruded sealer is essential in triggering side effects, CSBS has the advantage of having biocompatible ingredients without resin, which is supported by Riccuci et al., who demonstrated that the histologic section that extruded CSBS has no foreign body or inflammatory response [37]. The high pH of CSBS sealers may also provide several biological advantages, such as promoting hard tissue formation and interfering with osteoclastic activity, leading to favorable healing [26,36].

In this study, postoperative pain was rarely found in all the groups for the follow-up period (Table 3). The presence of preoperative pain is one of the predisposing factors to postoperative pain [21,38]; as such, symptomatic teeth were excluded to minimize the variables. Sealer-based obturation without the use pressure may be linked to rare postoperative pain. Indeed, the VAS score was slightly increased from 0.06 to 0.13 in the AH Plus group (Table 3). However, the other groups did not change, and neither percussion nor palpation revealed a significant response in all the groups. Our study is in line with many other studies in which the postoperative pain is associated with preoperative pain and sealer extrusion, not the type of sealer [21,38]. However, Ruparel et al. reported that, unlike CSBS, a low concentration AH plus sealer evoked calcitonin-gene-related peptide (CGRP) release [35]. This may be considered a cause of higher levels of postoperative pain.

Except for one case from the ADseal group, all the cases were effectively maintained for the short-term outcome up to three months. The failed case showed a sinus tract after a month, which originated from a cemental tear on the mesial root surface. Even after a month, the radiolucency of the AH Plus, ADseal, and EndoSeal groups had decreased in some cases. At 3-month recall, ERBS and CSBS exhibited similar radiographic changes in cases with preoperative periapical radiolucency, with 38.5% (5/13) and 33.3% (6/18), respectively. This study showed favorable short-term outcomes in both types of sealers. However, there are concerns about the hydraulic resorption of the calcium silicate sealers in some previous studies [8,25]. Nonetheless, our study is significant in that it presented the clinical efficacy of CSBS compared to ERBS. This study has limitations, including a small number of cases and a short-term evaluation. Therefore, long-term clinical studies are required to further evaluate this concern.

## 5. Conclusions

In conclusion of the present pilot clinical study, we found no significant differences in postoperative pain, void, and sealer extrusion among different CSBSs and ERBSs in 1-week, 1-month, and 3-month evaluations. Collectively, calcium-silicate-based sealer with sealer based obturation may provide comparable clinical efficiency to epoxy-resin-based sealer.

## Figures and Tables

**Table 1:** Case distribution.

	AH Plus (n = 16)	ADseal (n = 19)	CeraSeal (n = 17)	EndoSeal TCS (n = 20)
Sex				
Male	7	8	5	9
Female	9	11	12	11
Age				
≤30	2	2	3	1
30~60	5	7	7	13
61≤	9	10	7	6
Arch				
Maxilla	5	10	10	10
Mandible	11	9	7	10
Tooth type				
Anterior	3	5	4	1
Premolar	3	3	2	5
Molar	10	11	11	14

**Table 2:** Filling quality evaluation immediately after canal filling (n = 72).

	Void (%)		Sealer Extrusion (%)			
	Tooth	*p* Value	Tooth	*p* Value	Canal	*p* Value
AH Plus	56.3 (9/16)	0.145	56.3 (9/16)	0.067	33.3 (12/36)	0.139
ADseal	31.6 (6/19)		21.1 (4/19)		12.2 (5/41)	
CeraSeal	29.4 (5/17)		58.8 (10/17)		29.7 (11/37)	
EndoSeal	60.0 (12/20)		35.0 (7/20)		17.8 (8/45)	

**Table 3:** Postoperative pain after root canal filling (n = 71).

	VAS (Mean)			Percussion (+) (n)			Palpation (+) (n)		
	1-Week	1-Month	3-Month	1-Week	1-Month	3-Month	1-Week	1-Month	3-Month
AH Plus (16)	0.06	0.13	0.13	0	0	0	0	0	0
ADseal (18)	0	0	0	1	0	0	0	0	0
CeraSeal (17)	0	0	0	0	0	0	0	0	0
EndoSeal (20)	0.05	0.05	0.05	0	0	0	0	0	0

VAS: visual analog scales.

**Table 4:** Radiographic change (healing) of periapical lesion.

	Cases with Lesion	1-Week			1-Month			3-Month		
		Worse	C *	Healing	Worse	C *	Healing	Worse	C *	Healing
AH Plus	6	0	16	0	0	14	2	0	14	2
ADseal	7	0	18	0	0	16	2	0	15	3
CeraSeal	8	0	17	0	0	17	0	0	15	2
EndoSeal	10	0	20	0	0	18	2	0	16	4

* C: constant.

## Data Availability

The data presented in this study are available on request from the corresponding author. The data are not publicly available due to containing personal information.

## References

[1] Ørstavik D. (2014). Endodontic filling materials. Endod. Top..

[2] Trope M., Bunes A., Debelian G. (2015). Root filling materials and techniques: Bioceramics a new hope?. Endod. Top..

[3] Lim M., Jung C., Shin D.H., Cho Y.B., Song M. (2020). Calcium silicate-based root canal sealers: A literature review. Restor. Dent. Endod..

[4] Al-Haddad A., Che Ab Aziz Z.A. (2016). Bioceramic-based root canal sealers: A review. Int. J. Biomater..

[5] Horsted-Bindslev P., Andersen M.A., Jensen M.F., Nilsson J.H., Wenzel A. (2007). Quality of molar root canal fillings performed with the lateral compaction and the single-cone technique. J. Endod..

[6] Marciano M.A., Ordinola-Zapata R., da Cunha T.V., Duarte M.A.H., Cavenago B.C., Garcia R.B., Bramante C.M., Bernardineli N., Moraesl I.G. (2011). Analysis of four gutta-percha techniques used to fill mesial root canals of mandibular molars. Int. Endod. J..

[7] Somma F., Cretella G., Carotenuto M., Pecci R., Bedini R., De Biasi M., Angerame D. (2011). Quality of thermoplasticized and single point root fillings assessed by micro-computed tomography. Int. Endod. J..

[8] Poggio C., Arciola C.R., Dagna A., Colombo M., Bianchi S., Visai L. (2010). Solubility of root canal sealers: A comparative study. Int. J. Artif. Organs.

[9] Schweikl H., Schmalz G., Federlin M. (1998). Mutagenicity of the root canal sealer AH Plus in the Ames test. Clin. Oral Investig..

[10] Azar N.G., Heidari M., Bahrami Z.S., Shokri F. (2000). In vitro cytotoxicity of a new epoxy resin root canal sealer. J. Endod..

[11] Sousa C.J., Montes C.R., Pascon E.A., Loyola A.M., Versiani M.A. (2006). Comparison of the intraosseous biocompatibility of AH Plus, EndoREZ, and Epiphany root canal sealers. J. Endod..

[12] López-García S., Myong-Hyun B., Lozano A., García-Bernal D., Forner L., Llena C., Guerrero-Gironés J., Murcia L., Rodríguez-Lozano F.J. (2020). Cytocompatibility, bioactivity potential, and ion release of three premixed calcium silicate-based sealers. Clin. Oral Investig..

[13] Donnermeyer D., Burklein S., Dammaschke T., Schafer E. (2019). Endodontic sealers based on calcium silicates: A systematic review. Odontology.

[14] Abedi-Amin A., Luzi A., Giovarruscio M., Paolone G., Darvizeh A., Agulló V.V., Sauro S. (2017). Innovative root-end filling materials based on calcium-silicates and calcium-phosphates. J. Mater. Sci. Mater. Med..

[15] Kharouf N., Arntz Y., Eid A., Zghal J., Sauro S., Haikel Y., Mancino D. (2020). Physicochemical and antibacterial properties of novel, premixed calcium silicate-based sealer compared to powder-liquid bioceramic sealer. J. Clin. Med..

[16] Kim S.R., Kwak S.W., Lee J.K., Goo H.J., Ha J.H., Kim H.C. (2019). Efficacy and retrievability of root canal filling using calcium silicate-based and epoxy resin-based root canal sealers with matched obturation techniques. Aust. Endod. J..

[17] Faul F., Erdfelder E., Lang A.G., Buchner A. (2007). G*Power 3: A flexible statistical power analysis program for the social, behavioral, and biomedical sciences. Behav. Res. Methods.

[18] Fonseca B., Coelho M.S., Bueno C.E.dS., Fontana C.E., Martin A.S.D., Rocha D.G.P. (2019). Assessment of extrusion and postoperative pain of a bioceramic and resin-based root canal sealer. Eur. J. Dent..

[19] Ferreira N.S., Gollo E.K.F., Boscato N., Arias A., Silva E. (2020). Postoperative pain after root canal filling with different endodontic sealers: A randomized clinical trial. Braz. Oral Res..

[20] Guivarc’h M., Jeanneau C., Giraud T., Pommel L., About I., Azim A.A., Bukiet F. (2020). An international survey on the use of calcium silicate-based sealers in non-surgical endodontic treatment. Clin. Oral Investig..

[21] Yu Y.H., Kushnir L., Kohli M., Karabucak B. (2021). Comparing the incidence of postoperative pain after root canal filling with warm vertical obturation with resin-based sealer and sealer-based obturation with calcium silicate-based sealer: A prospective clinical trial. Clin. Oral Investig..

[22] Alonso-Ezpeleta L.O., Gasco-Garcia C., Castellanos-Cosano L., Martin-Gonzalez J., Lopez-Frias F.J., Segura-Egea J.J. (2012). Postoperative pain after one-visit root-canal treatment on teeth with vital pulps: Comparison of three different obturation techniques. Med. Oral Patol. Oral Circ. Bucal..

[23] Tan H.S.G., Lim K.C., Lui J.N., Lai W.M.C., Yu V.S.H. (2021). Postobturation pain associated with tricalcium silicate and resin-based sealer techniques: A randomized clinical trial. J. Endod..

[24] Parirokh M., Torabinejad M. (2010). Mineral trioxide aggregate: A comprehensive literature review—Part III: Clinical applications, drawbacks, and mechanism of action. J. Endod..

[25] Sfeir G., Zogheib C., Patel S., Giraud T., Nagendrababu V., Bukiet F. (2021). Calcium silicate-based root canal sealers: A narrative review and clinical perspectives. Materials.

[26] Lim E.S., Park Y.B., Kwon Y.S., Shon W.J., Lee K.W., Min K.S. (2015). Physical properties and biocompatibility of an injectable calcium-silicate-based root canal sealer: In vitro and in vivo study. BMC Oral Health.

[27] Chopra V., Davis G., Baysan A. (2021). Physico-chemical properties of calcium-silicate vs. resin based sealers-A systematic review and meta-analysis of laboratory-based studies. Materials.

[28] Santos-Junior A.O., Tanomaru-Filho M., Pinto J.C., Tavares K., Torres F.F.E., Guerreiro-Tanomaru J.M. (2021). Effect of obturation technique using a new bioceramic sealer on the presence of voids in flattened root canals. Braz. Oral Res..

[29] Penha da Silva P.J., Marceliano-Alves M.F., Provenzano J.C., Dellazari R.L.A., Gonçalves L.S., Alves F.R.F. (2021). Quality of root canal filling using a bioceramic sealer in oval canals: A three-dimensional analysis. Eur. J. Dent..

[30] Al-Jadaa A., Attin T., Peltomäki T., Heumann C., Schmidlin P.R., Paquè F. (2018). Influence of the internal anatomy on the leakage of root canals filled with thermoplastic technique. Clin. Oral Investig..

[31] Tanomaru-Filho M., Pinto J.C., Torres F.F.E., de Souza P.H.F., Pereira M.C., Guerreiro-Tanomaru J.M. (2020). Flow, filling ability and apical extrusion of new calcium silicate-based sealers: A micro-computed tomographic study. Dent. Oral Biol. Craniofac. Res..

[32] Ricucci D., Rocas I.N., Alves F.R., Loghin S., Siqueira J.F. (2016). Apically extruded sealers: Fate and influence on treatment outcome. J. Endod..

[33] Sari S., Duruturk L. (2007). Radiographic evaluation of periapical healing of permanent teeth with periapical lesions after extrusion of AH Plus sealer. Oral Surg. Oral Med. Oral Pathol. Oral Radiol. Endod..

[34] Scarparo R.K., Grecca F.S., Fachin E.V. (2009). Analysis of tissue reactions to methacrylate resin-based, epoxy resin-based, and zinc oxide-eugenol endodontic sealers. J. Endod..

[35] Ruparel N.B., Ruparel S.B., Chen P.B., Ishikawa B., Diogenes A. (2014). Direct effect of endodontic sealers on trigeminal neuronal activity. J. Endod..

[36] Gomes-Filho J.E., Watanabe S., Cintra L.T., Nery M.J., Dezan-Júnior E., Queiroz I.O., Lodi C.S., Basso M.D. (2013). Effect of MTA-based sealer on the healing of periapical lesions. J. Appl. Oral Sci..

[37] Ricucci D., Grande N.M., Plotino G., Tay F.R. (2020). Histologic response of human pulp and periapical tissues to tricalcium silicate-based materials: A series of successfully treated cases. J. Endod..

[38] Ali A., Olivieri J.G., Duran-Sindreu F., Abella F., Roig M., Garcia-Font M. (2016). Influence of preoperative pain intensity on postoperative pain after root canal treatment: A prospective clinical study. J. Dent..

